# Screening of hub genes and evaluation of the growth regulatory role of CD44 in metastatic prostate cancer

**DOI:** 10.3892/or.2022.8343

**Published:** 2022-06-06

**Authors:** Junhao Lin, Zhi Chen, Zuan Li, Deyong Nong, Ximing Li, Guihai Huang, Nan Hao, Jianbo Liang, Wei Li

Oncol Rep 46: 196, 2021; DOI: 10.3892/or.2021.8147

Subsequently to the publication of the above article, the authors have realized that they inadvertently included images of the same mice in Figs. 7A [the Negative Control (NC) experiment] and 8A [the 5B-3CT + Docetaxel (10 mg/kg) experiment].

After having consulted their original data, the authors have realized that these mice were correctly shown in the paper for the experiments portrayed in Fig. 7A; therefore, the corrected version of Fig. 8 is shown on the next page, showing the mice pertaining to the 5B-3CT + Docetaxel (10 mg/kg) experiment in Fig. 8A. The authors are grateful to the Editor of *Oncology Reports* for allowing them the opportunity to publish a Corrigendum, and all the authors agree to this Corrigendum. Furthermore, they apologize to the readership for any inconvenience caused.

## Figures and Tables

**Figure 8.**
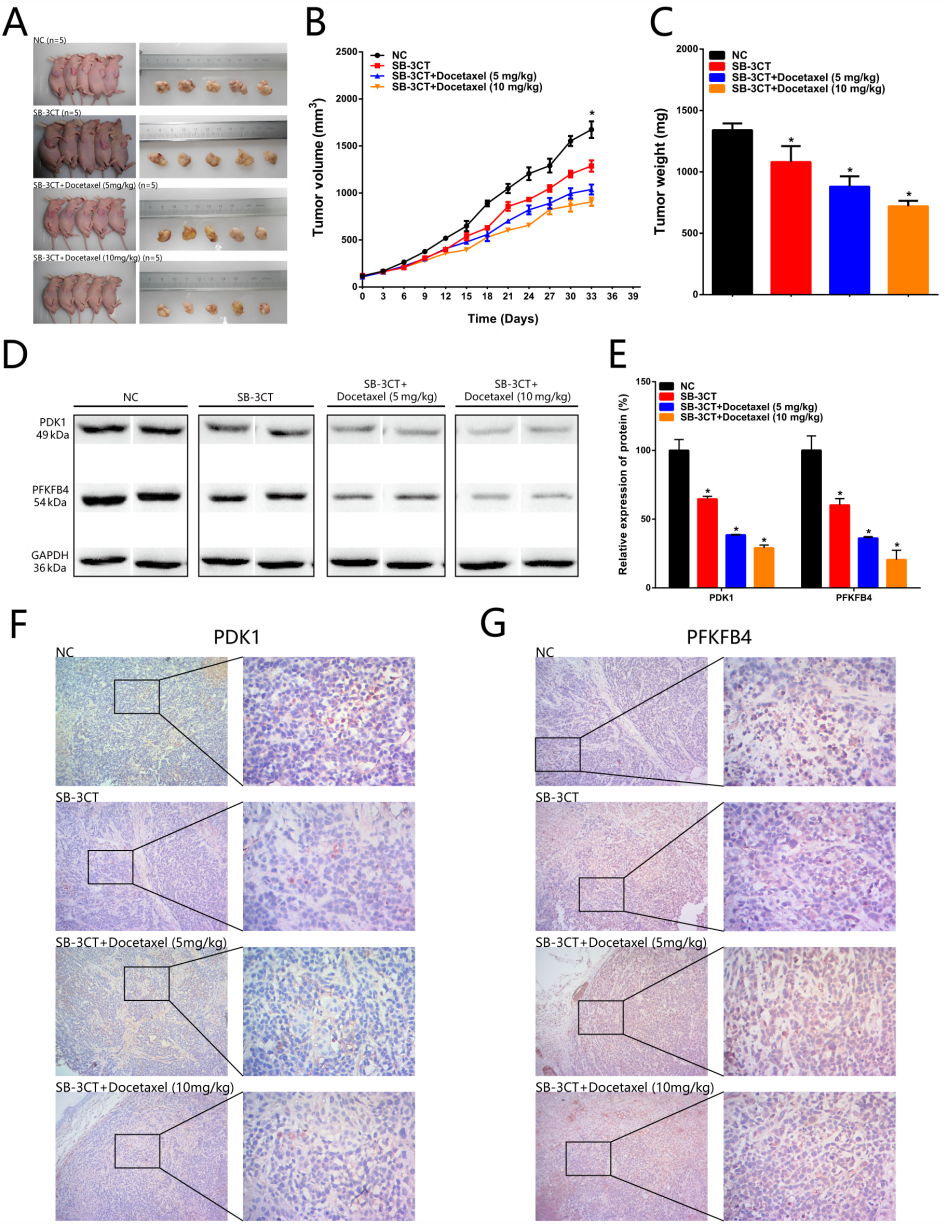
Inhibition of CD44 suppresses tumorigenicity of prostate cancer cells *in vivo* and the CD44 inhibitor (SB-3CT) combined with docetaxel inhibits tumorigenicity of prostate cancer. (A) Tumors dissected from BALB/c nude mice are presented (n=5 for each group). (B) Tumor volume curve of NC, SB-3CT, SB-3CT + Docetaxel (5 mg/kg) and SB-3CT + Docetaxel (10 mg/kg) treatment groups. (C) Tumor weight of NC, SB-3CT, SB-3CT + Docetaxel (5 mg/kg) and SB-3CT + Docetaxel (10 mg/kg) treatment groups. (D and E) PDK1 and PFKFB4 expression levels were downregulated in the PC-3 + SB-3T group, PC-3 + SB-3T + Docetaxel (5 mg/kg) group and PC-3 + SB-3T + Docetaxel (10 mg/kg) group compared with the PC-3 + NC group. (F) PDK1 expression levels were downregulated in the PC-3 + SB-3T group, PC-3 + SB-3T + Docetaxel (5 mg/kg) group and PC-3 + SB-3T + Docetaxel (10 mg/kg) group compared with the PC-3 + NC group. (G) PFKFB4 expression levels were downregulated in the PC-3 + SB-3T group, PC-3 + SB-3T + Docetaxel (5 mg/kg) group and PC-3 + SB-3T + Docetaxel (10 mg/kg) group compared with the PC-3 + NC group. Data are analysed using an ANOVA with Tukey's post hoc test. Data are presented as the mean ± SD. *P<0.05. NC, negative control; PDK1, pyruvate dehydrogenase kinase 1; PFKB4, 6-phosphofructo-2-kinase/fructose-2,6-biphosphatase 4.

